# 

**Published:** 1917-09-15

**Authors:** 


### CONTENTS.

| | PAGE |
|---|---|
| **A Campaign for Food Economy** ... ... ... | 479 |
| **Air-Raids on Hospitals and Non-Combatants** | 480 |
| **Hospital and Institutional News** ... ... ... | 481 |
| Institutions for Discharged Soldiers—The Medical Treatment of Invalided Soldiers—"The Soldier's Return"—A Fine Provincial Surgeon—Italian Medals for British Red Cross Workers—The late Mr. Clare Melhado—Temporary Appointments in War-time—Jubilee of the Metropolitan Asylums Board—Have the Hut-Hospitals come to Stay?—Liability for Sanatorium Treatment—Poor Law or Insurance Committee?—St. Katharine's in the East—The Swansea Hospital's Debt—The Hospital Problem in Cumberland—Mr. Hoult's Birthday Gifts—The New Ward at Morton Banks—The Status of Hornsey Cottage Hospital—Detectives in a Mental Hospital—A Bedroom Bazaar—Curious Conditional Gifts—Fruit, Fish and Vegetables—The Tribunals and the Hospitals—New Bread—Thirty Years of Poor-Law Service—The Increase of Prices—The Craigleith Quarterly—"Happy though Wounded"—The Problem of A 31—This Week's Drug Market. | |
| **Nerve-Shattered Soldiers and their Treatment** | 487 |
| Dr. Lumsden's Excellent Scheme. | |
| **The Work of the Local Government Board** ... | 488 |
| Far-reaching War Activities. | |
| **The Y.M.C.A. Anti-Tuberculosis Campaign** ... | 489 |
| An Educational Biograph Film. | |
| **Asylum Treatment: What is the Explanation?** | 490 |
| **Adequate Payment for Whole-Time Services** | 490 |
| The Bath Case and its Lessons. | |
| **The Editor's Letter-Box** ... ... ... ... | 491 |
| The Theatre and the Cinema: Some Hygienic Defects, their Causes and Prevention. A Children's Hospital in Difficulties. The British Nursing Profession: The Two Voices. | |
| **Nursing Affairs in Ireland**... ... ... ... | 492 |
| The Question of Economics. | |
| **The Matrons' and Sisters' Department** ... | 493 |
| Matrons in Council: VI. Modern Ideas Needed, though not Favoured in our Hospitals. | |
| Round the Hospitals. | |
| Nurse-sweating under Charity's Cloak—Economic Considerations: Two Points of View—The College's Vital Principle—Are Military Patients Under-fed?—The Editor and the Matrons—The Demand for Hospital Bags. | |
| **Some Problems of To-day**... ... ... ... | 496 |
| VI. Education and National Morality. | |
| **Matrons' and Sisters' Appointments** ... ... | 498 |
| **Cambridge and District Workers' Hospital Fund** | 498 |
| **The Institutional Worker** ... ... | 1 |

